# TVI_SARS-CoV-2_: a multicriteria index and urban dynamics in Minas Gerais, Brazil

**DOI:** 10.11606/s1518-8787.2025059006877

**Published:** 2026-01-09

**Authors:** Matheus Luiz Jorge Cortez, Úrsula Ruchkys de Azevedo, Sónia Maria Carvalho Ribeiro, Anacleto Marito Diogo, Danilo Cirino Muniz do Nascimento

**Affiliations:** I Universidade Federal de Minas Gerais. Instituto de Geociências. Belo Horizonte, MG, Brasil; II Universidade José Eduardo dos Santos. Huambo, Angola

**Keywords:** Demography, Epidemiology, Healthcare Disparities, Socioeconomic Factors

## Abstract

**OBJECTIVE:**

To develop a municipal-level vulnerability index to COVID-19 transmission that integrates socioeconomic factors, urban infrastructure, mobility, and composite indicators of social vulnerability and leisure for Minas Gerais. This study found factors associated with the spatial distribution of COVID-19 and classified the 853 municipalities in the state by vulnerability levels.

**METHODS:**

Data on COVID-19 cases from February 2022 were combined with nine variables in three domains: (i) socioeconomic factors (trade, service, construction, and manufacturing jobs); (ii) urban infrastructure and mobility (urban area, road connectivity, and vehicle density); and (iii) composite indicators (index of social vulnerability and access to culture, sports, and leisure). A multicriteria analysis model with Pearson’s correlations was elaborated and validated by cross-validation.

**RESULTS:**

The vulnerability index ranged from 1 to 8. The municipality of Belo Horizonte showed the highest value (8), followed by Uberlândia (6) and other medium to large municipalities with high urban dynamism. Urban area (22.92%), road connectivity (17.54%), and vehicle density (12.86%) constituted the most influential variables in the model. Spatial distribution indicated greater vulnerability in metropolitan regions and regional hubs.

**CONCLUSIONS:**

The proposed index highlighted the role of structural and occupational characteristics in territorial vulnerability to COVID-19. Limitations such as the time lag of some variables, source heterogeneity, and case underreporting—especially in municipalities with lower testing capacity—may have influenced the results. Incorporating emerging technologies and sustainable practices can mitigate the risks of future pandemics and improve quality of life. The index offered a useful tool for health planning (which may be adapted to other communicable diseases).

## INTRODUCTION

The COVID-19 pandemic surprised the world, imposing a true test of resilience on human societies. Its risk factors include an intrinsic element of modern life: permanent communities. Social and urban organization has played a crucial role in the spread of infectious diseases since the transition from hunter-gatherer to sedentary societies.

Socioeconomic inequalities significantly impact the health of the global population^1^. In the context of COVID-19, characteristics such as income, housing conditions, education, and occupation have been associated with clinical outcomes and spread rate^4^. Studies have also shown the role of urban structure and mobility in disease transmission^9^. Thus, understanding the spatial distribution of factors associated with COVID-19 is essential to find populations under greater susceptibility to the virus^12^.

The analysis of regional vulnerability has become a strategic instrument to guide mitigation actions and resource allocation in health emergencies. Several countries have used multicriteria decision analysis (MCDA) models, such as China^13^, India^14^, and Italy^15^, enabling the integration of social, economic, environmental, and territorial variables^13,16^. Several studies have used MCDA to find areas that are more prone to SARS-CoV-2 infection and to investigate factors associated with transmission^4,14,15^. In Brazil, a recent survey used MCDA to find priority areas for intervention against COVID-19 in Minas Gerais^4^. These studies offer relevant subsidies for the formulation of territory-sensitive public policies^19^.

Sustainable Development Goal 3 of the United Nations 2030 Agenda highlights how important it is to “ensure healthy lives and promote well-being for all at all ages”^23^. Its subitem “3.d” emphasizes the need to strengthen global capacity for early warning and management of health risks—a guideline in line with the challenges of the pandemic.

Minas Gerais, the second most populous state in Brazil, had an estimated population of 20,539,989 inhabitants across its 853 municipalities in 2022^24^. On July 28, 2022, the state recorded 3,809,396 confirmed cases of COVID-19 and 62,866 deaths due to it^25^, highlighting the seriousness of the health crisis and the importance of targeted strategies to face future pandemics.

In this scenario, despite ample production on the social and territorial determinants of COVID-19, gaps remain in the availability of instruments that can integrate multiple factors associated with the transmission of the disease on a municipal scale—especially under great socio-spatial diversity, such as Minas Gerais. The absence of synthetic indicators that simultaneously consider structural, economic, and mobility aspects hinders the identification of more susceptible territories and compromises the formulation of evidence-based responses. This requires an index that synthesizes elements that influence municipal vulnerability to COVID-19 in an integrated manner.

This study aims to develop a municipal vulnerability index to SARS-CoV-2 transmission in Minas Gerais by considering socioeconomic, urban infrastructure, and mobility variables. Its specific objectives involve (i) finding socioeconomic variables, urban infrastructure and mobility indicators, and composite indicators of social vulnerability and leisure with periodic updating and availability on a municipal scale that explain the distribution of COVID-19 cases, (ii) classifying its 853 municipalities according to their levels of vulnerability to the transmissibility of the virus, and (iii) examining the relative influence of these variables on territorial susceptibility to disease transmission. The spatialization of vulnerability levels can subsidize public policies to prevent and mitigate damages due to pandemics, increasing resilience in Minas Gerais.

## METHODS

The methodology in this study was structured in stages based on a MCDA model and according to its objectives. The independent variables were organized into three conceptual domains: (i) socioeconomic factors, (ii) urban infrastructure and mobility, and (iii) composite indicators of social vulnerability and leisure. The data, sources, selection criteria, and analytical procedures are detailed below.

### COVID-19 Data

Data on COVID-19 cases (dependent variable C-COV) were obtained at the municipal level from the Minas Gerais State Department of Health via the open case notification database available on its official website^26^. The database includes cases notified by laboratory, clinical, and epidemiological criteria.

February 2022 was chosen as the time frame of the analysis. Such choice stems from the significant increase in cases due to the highly transmissible Omicron variant in the period, characterizing an epidemiological peak that represents the pandemic in the state^27^. This period concentrated the largest volume of notifications, enabling us to observe transmission behavior at its recent peak without prolonged oscillations of external factors. This approach also provides methodological homogeneity, avoiding the dilution of data due to annual sums.

All 853 municipalities in Minas Gerais had notified cases of COVID-19 by February 2022, which enabled the MCDA model to be uniformly applied across the state. February 2022 showed the highest volume of monthly notifications since the beginning of the pandemic, corresponding to the phase of greatest transmissibility of COVID-19 in Minas Gerais (strongly influenced by the spread of the Omicron variant).

### Independent Variables: Conceptual Selection and Organization

The independent variables were chosen based on a literature review on factors associated with the spread of COVID-19 and social and territorial vulnerability, as per Benita et al.^28^ and Rocha et al.^29^, pointing to the relevance of indicators of inequality, occupation, and infrastructure in explaining territorial susceptibility to the pandemic. The variables are described below by conceptual domain:

i) Socioeconomic domain:

- SSJ (service sector jobs)
- TSJ (trade sector jobs)
- CIJ (construction industry jobs)
- MIJ (manufacturing industry jobs)

The choice of these variables considered their theoretical relevance in the literature and the availability and standardization of data on a municipal scale. This strategy consistently represented the structural, social, and mobility dimensions associated with territorial vulnerability to COVID-19.

These variables express the proportion of the population linked to sectors with high social interaction or agglomeration, which are considered more susceptible to the spread of the virus. The data were obtained from Fundação João Pinheiro^30^, referring to 2019. The values were transformed into percentages of the total population of each municipality.

ii) Urban Infrastructure and Mobility Domain:

- A-URB (urban area of municipalities) – obtained from the land cover raster of the 7.0 collection of MapBiomas^31^ by counting the pixels classified as “urban area”. The data were processed with a resolution of 30 meters per pixel. As per Yu et al.^32^ and Connolly et al.^9^, A-URB is directly associated with building density, urban sprawl, and population concentration, aspects that significantly contribute to the spread of respiratory diseases in dense urban contexts.
- A-COR (Corridor Area) – based on the vector files of the OpenStreetMap project^33^, including streets, avenues, and highways. The vectors were rasterized with a resolution of 10 meters, and the total area per municipality was calculated based on a pixel count.
- VD (vehicle density) – extracted from the Fundação João Pinheiro base (2019) and shown in vehicles per km^2^. It represents the intensity of individual transportation in the municipalities, associated with mobility patterns and emission of pollutants.

iii) Composite Indicators:

- The *Índice Mineiro de Responsabilidade Social – Vulnerabilidade* ^30,34^ (IMRS-V – Minas Gerais Social Responsibility Index – Vulnerability), developed by Fundação João Pinheiro, represents the social vulnerability of the municipalities of Minas Gerais. Its composition is based on socioeconomic variables, such as the proportion of people with an income below half a minimum wage, the percentage of households without access to piped water and sanitation, the demographic dependency ratio, among other indicators.
- The *Índice Mineiro de Responsabilidade Social – Cultura, Esporte e Lazer* ^30,34^ (IMRS-CEL – Minas Gerais Social Responsibility Index – Culture, Sport, and Leisure), developed by Fundação João Pinheiro, expresses the supply of culture, sports and leisure equipment and activities in the municipalities in Minas Gerais. The composition of the index includes variables such as the existence of public libraries, cultural centers, sports fields, leisure clubs, among others.

The use of composite indices is justified by their statistical validity, institutional recognition, and ability to represent multidimensionally phenomena. These indicators, widely used in diagnoses and public policies in Minas Gerais, use 2019 as their reference year since it is the most recent set available at the municipal level with statewide coverage and the sub-indices of interest.

Operationalizing the index on DINAMICA EGO^35^ required a linear transformation to convert the original values (represented by decimals) into integers, an essential condition for further raster processing.

### Multicriteria Analysis Model

The SARS-CoV-2 transmission vulnerability index (TVI_SARS-CoV-2_) was developed using a MCDA model on DINAMICA EGO^35^.

1. Attribution of scores to the independent variables: the values were classified into 11 ranges based on the maximum value of each variable divided by 10. Each municipality received a score from 1 to 10 (a 0 was assigned to cases with a value equal to zero).
2. Weighting of the variables: each score was multiplied by a weight that was calculated based on Pearson’s correlation between the independent variable and the number of COVID-19 cases (C-COV). The relative contribution of each variable was then shown as the percentage of its coefficient in relation to the summed total. These percentages were converted to a scale from 0 to 1 that divided the values by 100. The result generated a vector of weights with a sum totals equal to 1 that ensured proportionality between the variables in the index aggregation stage.
3. Calculation of the final index: the weighted sum of the scores was obtained.

The general formula of the index was:


$$TVI_{SARS-CoV-2}=\sum_{i=1}^{n}\left(p_{i}\times x_{i}\right)$$

In which:

•*p*_*i*_ represents the weight assigned to the variable *i*;
•*x*_*i*_ is the variable grade *i* at the municipal level;
•*n* is the total number of variables.

The sum of the weights was normalized to 1. The final index, which varied from 0 to 10, expresses the degree of municipal vulnerability to the transmission of COVID-19.

### Cross-validation

The MCDA model was validated using the k-fold cross-validation technique and the *scikit-learn* library in a Python environment^36^. The dataset was divided into k subsets. The model was trained on k−1 parts and tested on the remaining part. This process was repeated k times. The mean of the mean squared errors in each round was used as a performance metric. This approach ensures the generalization of the model^36^and avoids overfitting the input data.

## RESULTS

The analyzed variables showed several degrees of correlation with the number of new cases of COVID-19 in February 2022. Based on the intensity of these correlations, the MCDA weights were calculated to reflect the relevance of each variable in the model. Table 1 details the weights assigned to each variable.

**Table 1:** Values of Pearson’s correlations with the dependent variable C-COV, percentage corresponding to each variable according to the sum of the correlation values, and weights calculated for each variable.

Variable	Pearson^a^	Percentage^b^	Weight
A-URB	0.925	22.92	0.2292
A-COR	0.708	17.54	0.1754
SSJ	0.323	8.00	0.0800
TSJ	0.476	11.79	0.1179
CIJ	0.238	5.90	0.0590
MIJ	0.115	2.85	0.0285
IMRS-V	0.393	9.74	0.0974
IMRS-CEL	0.339	8.40	0.0840
VD	0.519	12.86	0.1286
Total	4.036	100	1

A-URB: urban area of the municipalities; A-COR: area of road corridors (streets and highways); SJ: service sector jobs; TSJ: trade sector jobs; CIJ: construction industry jobs; MIJ: manufacturing industry jobs; IMRS-V: Minas Gerais Social Responsibility Index – Vulnerability; IMRS-CEL: Minas Gerais Index of Social Responsibility – Culture, Sports, and Leisure; VD: vehicle density.

^a^ Significant values at the level of 1% probability.

^b^ Percentage of the variable correlation value relative to the sum of all values.

A descriptive analysis of the selected independent variables (which aimed to evince their statistical distribution across the 853 municipalities of Minas Gerais) preceded the application of the MCDA model. Table 2 shows the mean, standard deviations, medians, minimums, and maximums for each variable, evincing the structural and socioeconomic heterogeneity of the state.

**Table 2:** Descriptive statistics of the independent variables to compose the TVISARS-CoV-2.

Variable	Mean	Standard deviation	Median	Minimum	Maximum
A-URB	5.2	15.1	1.57	0	287.41
A-COR	5.37	6.07	3.37	0.25	64.42
SSJ	7.09	3.97	6.34	0.21	74.78
TSJ	2.37	1.68	1.97	0.17	12.08
CIJ	0.39	1.03	0.09	0	11.47
MIJ	2.52	4.09	0.97	0	44.2
IMRS-V	0.54	0.1	0.54	0.29	0.82
IMRS-CEL	0.53	0.19	0.52	0	1
VD	38.22	256.28	10.21	0.16	6,902.97

TVI_SARS-CoV-2_: vulnerability index to transmission of the SARS-CoV-2 virus; A-URB: urban area of the municipalities; A-COR: area of road corridors (streets and highways); SSJ: service sector jobs; TSJ: trade sector jobs; CIJ: construction industry jobs; MIJ: manufacturing industry jobs; IMRS-V: Minas Gerais Social Responsibility Index – Vulnerability; IMRS-CEL: Minas Gerais Index of Social Responsibility – Culture, Sports, and Leisure; VD: vehicle density.

**Figure 1:**
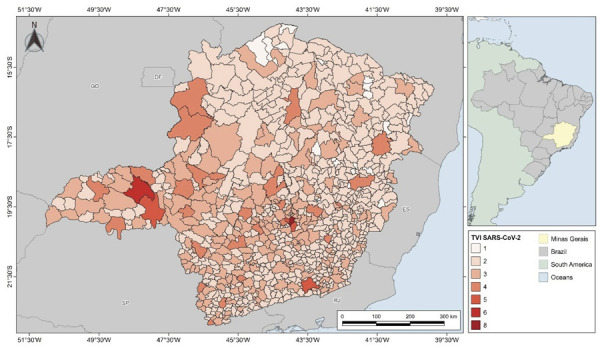
TVISARS-CoV-2 levels for the municipalities of Minas Gerais.

The TVI_SARS-CoV-2_ obtained by the multicriteria analysis model ranged from 1 to 8 for the 853 municipalities of Minas Gerais (Figure 1). The municipalities that obtained a 1 showed the lowest risk of transmission of the disease. On the other hand, this study classified those with higher TVI_SARS-CoV-2_ values as under a higher risk of viral transmission.

The municipality of Belo Horizonte showed the highest TVI_SARS-CoV-2_ value (8), followed by Uberlândia (6). In total, four other municipalities had an index of 5: Uberaba (adjacent to Uberlândia), Contagem and Nova Lima (bordering the capital), and Juiz de Fora (in southeastern Minas Gerais). The spatial distribution of the highest indices showed a concentration of high vulnerability in urban centers and metropolitan regions, such as the Metropolitan Region of Belo Horizonte, Triângulo Mineiro, Sul de Minas, and Zona da Mata. These territories have high urban density, road connectivity, and economic sectors with high social interaction—aspects reflected in the variables A-URB, A-COR, VD, SSJ, and TSJ, which are more important in the model. In turn, municipalities in northern and northeastern Minas Gerais (marked by lower population density and reduced urban and economic infrastructure) showed the lowest index values.

Of the 853 municipalities in Minas Gerais, 31 stood out for their highest levels of vulnerability to SARS-CoV-2 transmissibility. In addition to the six municipalities above (the indices of which ranged from 5 to 8), 25 other municipalities showed a TVI_SARS-CoV-2_ of 4 (Table 3). These municipalities are distributed across regions of the state, reflecting the heterogeneity of vulnerability to COVID-19 in Minas Gerais.

**Table 3:** Municipalities with the highest TVISARS-CoV-2 values according to the proposed model.

IBGE Code	Municipality	TVI	IBGE Code	Municipality	TVI
3106200	Belo Horizonte	8	3152501	Pouso Alegre	4
3170206	Uberlândia	6	3104007	Araxá	4
3170107	Uberaba	5	3134202	Ituiutaba	4
3118601	Contagem	5	3127701	Governador Valadares	4
3136702	Juiz de Fora	5	3127107	Frutal	4
3144805	Nova Lima	5	3148103	Patrocínio	4
3143302	Montes Claros	4	3147006	Paracatu	4
3148004	Patos de Minas	4	3131307	Ipatinga	4
3106705	Betim	4	3131703	Itabira	4
3170404	Unaí	4	3120904	Curvelo	4
3122306	Divinópolis	4	3147907	Passos	4
3103504	Araguari	4	3107406	Bom Despacho	4
3170701	Varginha	4	3126109	Formiga	4
3151800	Poços de Caldas	4	3168606	Teófilo Otoni	4
3167202	Sete Lagoas	4	3152105	Ponte Nova	4
3125101	Extreme	4	-	-	-

TVI: index of vulnerability to viral transmission; IBGE: *Instituto Brasileiro de Geografia e Estatística*.

**Figure 2:**
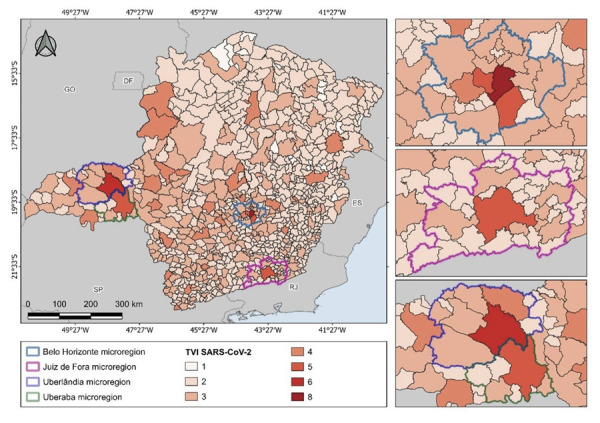
TVISARS-CoV-2 levels and micro-regions of greater vulnerability in Minas Gerais.

Figure 2 shows the aggregate regional vulnerability and the microregions with a higher TVI_SARS-CoV-2_ pattern. Microregions such as Belo Horizonte, Uberlândia, Uberaba, and Juiz de Fora strongly showed urban dynamism and regional mobility as risk factors. Moreover, some municipalities with high TVI_SARS-CoV-2_ also house regional health superintendencies or managements^40^, reinforcing their role as centers that articulate territorial flows. Thus, the spatialization of the index shows a logic of dissemination associated with urban centrality and the local network structure, aspects that should be considered when devising strategies to prevent and respond to future pandemics.

The model (evaluated by the mean squared errors in each iteration of the cross-validation) showed a quite satisfactory performance, according to its extremely small value: about 5.8331 x 10^32^.

## DISCUSSION

A-URB showed the greatest weight in the MCDA, contributing to 22.92% of its total weight. As in Table 1, this variable had the highest correlation with the number of COVID-19 cases (r = 0.925). This indicator is directly associated with the density of buildings and population concentration^41^. The growth of urban areas tends to increase the exposure of populations to respiratory diseases due to the greater proximity between individuals and the complexity of social networks^32,42^.

Moreover, urban centers attract regional, national, and international flows due to the offer of services and economic activities^43^. Such urban dynamism can generate scenarios that are more conductive to the spread of viruses, especially in places with unequal access to health and precarious housing conditions^9,47^.

The A-COR weight corresponded to 17.54% of the TVI_SARS-CoV-2_ (the second most important variable for the MCDA). Initially, the increase in COVID-19 cases occurred by hierarchical diffusion. Diffusion by contagion acquired greater relevance after the local transmission of the COVID-19 virus^48,49^. The movement of individuals infected by SARS-CoV-2 played a crucial role in increasing cases^50^. Moreover, population displacement by public transportation proved itself a relevant factor for viral transmissibility due to the agglomeration of people and exposure time^53,54^.

The higher connectivity and population density in urban areas tend to facilitate the rapid spread of pathogens such as SARS-CoV-2, whereas rural areas, although less dense, may be vulnerable due to connectivity to urban centers. Moreover, control measures may have limited effectiveness in urban areas when compared to rural ones^55^. In general, population density and intense social interactions in urban centers significantly contribute to the rapid spread of pathogens, increasing the risk of outbreaks and pandemics^56,57^.

Also related to mobility, VD had a weight (12.86%) in the model. In addition to the displacement of people, this variable has important environmental implications. The emission of air pollutants, such as that of particulate matter smaller than 2.5 and 10 micrometres^58,59^, can compromise individuals’ respiratory system and aggravate COVID-19 infections^60,61^. Studies have linked air pollution to the increase in cases and deaths from COVID-19 in several regions of the world^62^.

Regarding the occupational structure of the municipalities, TSJ and SSJ stood out, with weights of 11.79% and 8%, respectively. These sectors imply greater social interaction and crowding, which increases the risk of viral transmission (Table 1). Workers in these sectors (especially in essential services such as transportation, food, and health) have been particularly vulnerable during the pandemic^67^.

The IMRS-V and IMRS-CEL subindices corresponded to 9.74% and 8.40% of the weights of the MCDA, respectively. Thus, social vulnerabilities are positively related to higher incidences of COVID-19^5,7,70^. Beyond socioeconomic aspects, municipalities with a greater infrastructure for recreational purposes may have gathered people to the detriment of social distancing^68^. Moreover, the time of registration of the COVID-19 cases in this study (February 2022) included a certain tranquility and return to normality from a portion of the population due to the advance of vaccination campaigns^71^. Furthermore, a portion of society disregarded the great potential for dissemination of the Omicron variant (which was circulating in the state)^27^. Note that Ordinance GM/MS no. 913, of April 22, 2022, (which entered into force 30 days after its publication)^72^ terminated the status of “Public Health Emergency of National Importance as a result of human infection by the new coronavirus (2019-nCoV).”

In general, the variables in the model reflect the multiplicity of factors associated with territorial vulnerability to COVID-19 in Minas Gerais. The spatial distribution of TVI_SARS-CoV-2_ values shows the spread of the virus due to local urbanization, mobility, and the social structure of the municipalities.

The low errors obtained during cross-validation indicate the statistical consistency and robustness of the results. The index proved itself sensitive to intrastate variations, evincing territorial patterns of vulnerability to SARS-CoV-2 transmission, strengthening its potential for application in other communicable diseases and strategies to prevent future pandemics.

However, some limitations related to the independent variables stand out. Most of the socioeconomic and structural data refer to 2019 as it is the most recent set available at the municipal level with statewide coverage. This time lag in relation to the analyzed period (February 2022) may have partially compromised the timeliness of the estimates, especially in the face of the social and economic changes accelerated by the pandemic. Moreover, although this study chose its variables based on the consolidated literature, these proxies fail to directly capture more dynamic aspects, such as daily population flows or behavioral changes induced by public policies. Another point to consider is underreporting in Brazil, which can affect the quality and completeness of the used records^73^. Finally, the data came from several sources (such as Fundação João Pinheiro, MapBiomas, and OpenStreetMap) that use different scope and periodicity methodologies, which can introduce explanatory variable heterogeneity.

## FINAL CONSIDERATIONS

This study evaluated the vulnerability of municipalities in Minas Gerais to SARS-CoV-2 transmission using TVI_SARS-CoV-2_ (based on a MCDA model). The index highlighted the crucial role of socioeconomic and urban infrastructure variables (such as mobility, vehicle density, connectivity, and professional occupation) in the spread of the virus. Although these variables explain a portion of the transmission dynamics, limitations such as case underreporting and the absence of behavioral factors affect the accuracy of the model.

Despite limitations, such case underreporting and the absence of behavioral variables, TVI_SARS-CoV-2_ constitutes an appropriate tool to subsidize public policies, prioritize resources, and mitigate health crises. It can also be applied to other communicable diseases and adapted to other regional contexts, providing specific analyses aligned with local needs.

The results highlight the importance of understanding the socio-spatial dynamics that influence the vulnerability of territories, contributing to more efficient strategies to cope with pandemics. Our results innovatively show the need to include corridors and urban connectivity in territorial planning as a strategy to mitigate pandemic events. Future studies that explore the inclusion of behavioral variables and new methodological approaches can further improve the analyses in this study, increasing their applicability and accuracy.

State and municipal health contingency plans and territorial planning initiatives can incorporate the proposed index as a technical input to mitigate epidemiological risks.

## Data Availability

The data in this study are available upon request to the corresponding author.
