# Supplementary material for: TVISARS-CoV-2: a multicriteria index and urban dynamics in Minas Gerais, Brazil
Source: Rev Saude Publica. 2026 Jan 9;59:e49. doi: 10.11606/s1518-8787.2025059006877 (PMC12713526; doi:10.11606/s1518-8787.2025059006877)
Supplement: Translation

# Translation: IVT_SARS-CoV-2_: um índice multicritério e a dinâmica urbana em Minas Gerais, Brasil

**Authors:** Matheus Luiz Jorge Cortez, Úrsula Ruchkys de Azevedo, Sónia Maria Carvalho Ribeiro, Anacleto Marito Diogo, Danilo Cirino Muniz do Nascimento

**Affiliations:** I Universidade Federal de Minas Gerais. Belo Horizonte, Minas Gerais, Brasil; II Universidade José Eduardo dos Santos. Huambo, Angola

**Keywords:** Demografia, Epidemiologia, Disparidades em Assistência à Saúde, Fatores Socioeconômicos

## Abstract

**OBJETIVO:**

Desenvolver um índice de vulnerabilidade municipal à transmissão do SARS-CoV-2 em Minas Gerais, integrando fatores socioeconômicos, infraestrutura urbana, mobilidade e indicadores compostos de vulnerabilidade social e lazer. Identificaram-se os fatores associados à distribuição espacial da covid-19 e classificaram-se os 853 municípios do estado por níveis de vulnerabilidade.

**MÉTODOS:**

Foram utilizados dados de casos de covid-19 de fevereiro de 2022, combinados a nove variáveis organizadas em três domínios: (i) fatores socioeconômicos (empregos nos setores de comércio, serviços, construção e transformação); (ii) infraestrutura urbana e mobilidade (área urbana, conectividade rodoviária e densidade de veículos); e (iii) indicadores compostos (índice de vulnerabilidade social e de acesso à cultura, esporte e lazer). Aplicou-se um modelo de análise multicritério, com pesos definidos pelas correlações de Pearson. O índice final foi validado por validação cruzada.

**RESULTADOS:**

O índice de vulnerabilidade variou de 1 a 8. Belo Horizonte registrou o maior valor (8), seguida de Uberlândia (6) e outros municípios de médio a grande porte com elevado dinamismo urbano. As variáveis mais influentes no modelo foram área urbana (22,92%), conectividade rodoviária (17,54%) e densidade de veículos (12,86%). A distribuição espacial indicou maior vulnerabilidade em regiões metropolitanas e polos regionais.

**CONCLUSÕES:**

O índice destacou o papel de características estruturais e ocupacionais na vulnerabilidade territorial à covid-19. Limitações como a defasagem temporal de algumas variáveis, a heterogeneidade entre fontes e a subnotificação de casos — especialmente em municípios com menor capacidade de testagem — podem ter influenciado os resultados. A incorporação de tecnologias emergentes e práticas sustentáveis pode mitigar riscos de futuras pandemias e promover melhor qualidade de vida. Ainda assim, o índice demonstrou ser uma ferramenta útil ao planejamento sanitário, com potencial de adaptação a outras doenças transmissíveis.

## INTRODUÇÃO

A pandemia de covid-19 surpreendeu o mundo, impondo um verdadeiro teste de resiliência às sociedades humanas em diferentes contextos. Entre os fatores de risco associados à doença, destaca-se um elemento intrínseco à vida moderna: a convivência em comunidades permanentes. Desde a transição das sociedades caçadoras-coletoras para as sedentárias, a organização social e urbana tem desempenhado um papel crucial na disseminação de doenças infecciosas.

As desigualdades socioeconômicas têm um impacto significativo na saúde da população global^1^. No contexto da covid-19, características como renda, condições de moradia, escolaridade e ocupação têm sido associadas a desfechos clínicos e à taxa de disseminação do vírus^4^. Estudos também evidenciam o papel da estrutura urbana e da mobilidade na transmissão de doenças^9^. Portanto, compreender a distribuição espacial de fatores associados à covid-19 é essencial para identificar populações mais susceptíveis ao vírus^12^.

A análise da vulnerabilidade regional tornou-se um instrumento estratégico para orientar ações de mitigação e alocação de recursos em emergências sanitárias. Modelos de análise de decisão multicritério (ADMC) têm sido amplamente empregados em diferentes países, como China^13^, Índia^14^ e Itália^15^, permitindo a integração de variáveis sociais, econômicas, ambientais e territoriais^13,16^. Diversos estudos utilizaram ADMC para identificar áreas mais propensas à infecção pelo SARS-CoV-2 e para investigar os fatores associados à transmissão^4,14,15^. No Brasil, uma pesquisa recente empregou a ADMC para identificar áreas prioritárias de intervenção contra a covid-19 em Minas Gerais^4^. Esses estudos oferecem subsídios relevantes para a formulação de políticas públicas sensíveis ao território^19^.

A Agenda 2030 da Organização das Nações Unidas (ONU) destaca, por meio do Objetivo de Desenvolvimento Sustentável 3, a importância de “garantir o acesso à saúde de qualidade e promover o bem-estar para todos, em todas as idades”^23^. O subitem “3.d” enfatiza a necessidade de reforçar a capacidade global para alerta precoce e gerenciamento de riscos à saúde — diretriz alinhada aos desafios impostos pela pandemia.

Minas Gerais, o segundo estado mais populoso do Brasil, contava com uma população estimada de 20.539.989 habitantes em 2022, distribuída por seus 853 municípios^24^. Em 28 de julho de 2022, o estado registrou 3.809.396 casos confirmados de covid-19 e 62.866 óbitos decorrentes da doença^25^, evidenciando a gravidade da crise sanitária e a importância de estratégias direcionadas para enfrentar pandemias futuras.

Nesse cenário, embora haja ampla produção sobre os determinantes sociais e territoriais da covid-19, persistem lacunas quanto à disponibilidade de instrumentos capazes de integrar múltiplos fatores associados à transmissão da doença em escala municipal – sobretudo em contextos de grande diversidade socioespacial, como o de Minas Gerais. A ausência de indicadores sintéticos que considerem simultaneamente aspectos estruturais, econômicos e de mobilidade dificulta a identificação de territórios mais suscetíveis e compromete a formulação de respostas baseadas em evidências. Diante disso, torna-se necessário desenvolver um índice que sintetize, de forma integrada, elementos que influenciam a vulnerabilidade municipal à covid-19.

Este trabalho tem como objetivo desenvolver um índice de vulnerabilidade municipal à transmissão do SARS-CoV-2 em Minas Gerais, considerando variáveis socioeconômicas, de infraestrutura urbana e mobilidade. Os objetivos específicos são: (i) identificar variáveis socioeconômicas, indicadores de infraestrutura urbana e mobilidade, bem como indicadores compostos de vulnerabilidade social e lazer, todos com atualização periódica e disponibilidade em escala municipal, que expliquem a distribuição de casos de covid-19; (ii) classificar os 853 municípios do estado segundo seus níveis de vulnerabilidade à transmissibilidade do vírus; e (iii) examinar a influência relativa dessas variáveis na susceptibilidade territorial à transmissão da doença. A espacialização dos níveis de vulnerabilidade poderá subsidiar políticas públicas voltadas à prevenção e à mitigação de danos causados por pandemias, promovendo maior resiliência do território mineiro.

## MÉTODOS

A metodologia foi estruturada em etapas, conforme os objetivos do estudo, com base em um modelo ADMC. As variáveis independentes foram organizadas em três domínios conceituais: (i) fatores socioeconômicos, (ii) infraestrutura urbana e mobilidade, e (iii) indicadores compostos de vulnerabilidade social e lazer. A seguir, detalham-se dados, fontes, critérios de seleção e procedimentos analíticos empregados.

### Dados da covid-19

Os dados de casos de covid-19 (variável dependente C-COV) foram obtidos em escala municipal junto à Secretaria de Estado de Saúde de Minas Gerais (SES-MG), por meio da base de dados abertos de notificação de casos disponíveis no site oficial do governo estadual^26^. A base inclui casos notificados por critério laboratorial, clínico e epidemiológico.

Foi selecionado o mês de fevereiro de 2022 como recorte temporal da análise. A escolha se deve ao expressivo aumento de casos impulsionado pela variante Ômicron, altamente transmissível, o que caracteriza um pico epidemiológico representativo da pandemia no estado^27^. Esse período concentrou o maior volume de notificações, permitindo observar o comportamento da transmissão em seu ápice recente, sem oscilação prolongada de fatores externos. Esse recorte também proporciona homogeneidade metodológica, evitando a diluição dos dados causada por somatórios anuais.

É importante destacar que até fevereiro de 2022, todos os 853 municípios de Minas Gerais registraram casos notificados de covid-19. Esse cenário permitiu que o modelo ADMC fosse aplicado uniformemente em todo o estado. Fevereiro de 2022 se destacou como o mês com maior volume de notificações mensais desde o início da pandemia. Este período corresponde à fase de maior transmissibilidade da covid-19 em Minas Gerais, fortemente influenciada pela disseminação da variante Ômicron.

### Variáveis Independentes: Seleção e Organização Conceitual

As variáveis independentes foram selecionadas com base em revisão da literatura sobre fatores associados à disseminação da covid-19 e à vulnerabilidade social e territorial, conforme discutido por Benita et al.^28^ e Rocha et al.^29^, que apontam a relevância de indicadores de desigualdade, ocupação e infraestrutura na explicação da suscetibilidade territorial à pandemia. A seguir, as variáveis são apresentadas por domínio conceitual:

i) Domínio socioeconômico:

- EFS (Empregos no setor de serviços);
- EFC (Empregos no setor de comércio);
- EFIC (Empregos na indústria da construção);
- EFIT (Empregos na indústria de transformação).

A escolha dessas variáveis considerou, além da relevância teórica reconhecida na literatura, a disponibilidade e a padronização dos dados em escala municipal. Essa estratégia permitiu representar de forma consistente as dimensões estruturais, sociais e de mobilidade associadas à vulnerabilidade territorial à covid-19.

Essas variáveis expressam a proporção da população vinculada a setores com alta interação social ou aglomeração, considerados mais suscetíveis à disseminação do vírus. Os dados foram obtidos junto à Fundação João Pinheiro^30^ (FJP), referentes ao ano de 2019. Os valores foram transformados em percentual da população total de cada município.

ii) Domínio Infraestrutura Urbana e Mobilidade:

- A-URB (Área urbana dos municípios) – obtida a partir do raster de cobertura do solo da coleção 7.0 do MapBiomas^31^, pela contagem de pixels classificados como “área urbana”. Os dados foram processados com resolução de 30 metros por pixel. Conforme discutido por Yu et al.^32^ e Connolly et al.^9^, a variável A-URB está diretamente associada à densidade construtiva, à expansão urbana e à concentração populacional, aspectos que contribuem significativamente para a difusão de doenças respiratórias em contextos urbanos densos.
- A-COR (Área de corredores viários) – baseada nos arquivos vetoriais do projeto OpenStreetMap^33^, incluindo ruas, avenidas e rodovias. Os vetores foram rasterizados com resolução de 10 metros, e a área total por município foi calculada com base na contagem de pixels.
- DV (Densidade de veículos) – extraída da base da FJP (2019) e expressa em veículos por km^2^. Representa a intensidade do uso de transporte individual nos municípios, associada a padrões de mobilidade e emissão de poluentes.

iii) Domínio de Indicadores Compostos:

- O Índice Mineiro de Responsabilidade Social – Vulnerabilidade^30,34^ (IMRS-V), desenvolvido pela FJP, representa a vulnerabilidade social dos municípios mineiros. Sua composição é baseada em variáveis socioeconômicas, como a proporção de pessoas com renda inferior a meio salário mínimo, o percentual de domicílios sem acesso a água encanada e saneamento, bem como a razão de dependência demográfica, entre outros indicadores.
- O Índice Mineiro de Responsabilidade Social – Cultura, Esporte e Lazer^30,34^ (IMRS-CEL), desenvolvido pela FJP, expressa o nível de oferta de equipamentos e atividades voltadas à cultura, ao esporte e ao lazer nos municípios mineiros. A composição do índice inclui variáveis como a existência de bibliotecas públicas, centros culturais, campos poliesportivos e clubes de lazer, entre outras.

A utilização de índices compostos justifica-se por sua validade estatística, reconhecimento institucional e capacidade de representar fenômenos de maneira multidimensional. Esses indicadores, amplamente empregados em diagnósticos e políticas públicas em Minas Gerais, têm 2019 como ano de referência, por ser o conjunto mais recente disponível em nível municipal, com abrangência estadual e contemplando os subíndices de interesse.

Para a operacionalização do índice no ambiente DINAMICA EGO^35^, foi necessária a realização de uma transformação linear, a fim de converter os valores originais – representados por decimais – em números inteiros, condição essencial para o processamento raster posterior.

### Modelo de Análise Multicritério

O índice de vulnerabilidade à transmissão do SARS-CoV-2 (IVT_SARS-CoV-2_) foi desenvolvido por meio de um modelo ADMC no software DINAMICA EGO^35^.

1. Atribuição de notas às variáveis independentes: os valores foram classificados em 11 faixas com base no valor máximo de cada variável dividido por dez. Cada município recebeu uma nota de 1 a 10 (nota 0 foi atribuída aos casos com valor igual a zero).
2. Ponderação das variáveis: cada nota foi multiplicada por um peso, calculado com base na correlação de Pearson entre a variável independente e o número de casos de covid-19 (C-COV). A contribuição relativa de cada variável foi então expressa como o percentual de seu coeficiente em relação ao total somado. Esses percentuais foram convertidos para uma escala entre 0 e 1, dividindo-se os valores por 100. O resultado desse processo gerou um vetor de pesos cuja soma totaliza 1, assegurando a proporcionalidade entre as variáveis na etapa de agregação do índice.

Cálculo do índice final: foi obtida a soma ponderada das notas.

A fórmula geral do índice foi:


$$IVT_{\text{SARS-CoV-2 }}=\sum_{i=1}^{n}\left(p_{i}\times x_{i}\right)$$

Em que:

- *p*_*i*_ representa o peso atribuído à variável *i*;
- *x*_*i*_ é a nota da variável *i* no âmbito municipal;
- *n* é o número total de variáveis consideradas.

A soma dos pesos foi normalizada para 1. O índice final, com potencial de variação entre 0 e 10, expressa o grau de vulnerabilidade municipal à transmissão da covid-19.

### Validação Cruzada

A validação do modelo ADMC foi realizada por meio da técnica de validação cruzada k-fold, utilizando a biblioteca *scikit-learn* em ambiente Python^36^. O conjunto de dados foi dividido em k subconjuntos; o modelo foi treinado em k-1 partes e testado na parte restante, repetindo-se esse processo k vezes. A média dos erros quadráticos médios (EQM) obtidos em cada rodada foi usada como métrica de desempenho. Essa abordagem visa garantir a generalização do modelo^36^e evitar o sobreajuste aos dados de entrada.

## RESULTADOS

As variáveis analisadas apresentaram diferentes graus de correlação com o número de novos casos de covid-19 registrados em fevereiro de 2022. Com base na intensidade dessas correlações, os pesos para o ADMC foram calculados, de modo a refletir a relevância de cada variável no modelo. Os detalhes dos pesos atribuídos a cada variável estão apresentados na Tabela 1.

**Tabela 1:** Valores das correlações de Pearson com a variável dependente C-COV, percentual correspondente a cada variável de acordo com a soma dos valores da correlação e pesos calculados para cada variável.

Variável	Pearson^a^	Percentagem^b^	Peso
A-URB	0,925	22,92	0,2292
A-COR	0,708	17,54	0,1754
EFS	0,323	8,00	0,0800
EFC	0,476	11,79	0,1179
EFIC	0,238	5,90	0,0590
EFIT	0,115	2,85	0,0285
IMRS-V	0,393	9,74	0,0974
IMRS-CEL	0,339	8,40	0,0840
DV	0,519	12,86	0,1286
Total	4,036	100	1

A-URB: área urbana dos municípios; A-COR: área de corredores viários (ruas e rodovias); EFS: empregos no setor de serviços; EFC: empregos no setor de comércio; EFIC: empregos na indústria da construção; EFIT: empregos na indústria de transformação; IMRS-V: Índice Mineiro de Responsabilidade Social – Vulnerabilidade; IMRS-CEL: Índice Mineiro de Responsabilidade Social – Cultura, Esporte e Lazer; DV: densidade de veículos.

^a^ Valores significativos ao nível de 1% de probabilidade.

^b^ Percentagem do valor de correlação da variável em relação à soma de todos os valores.

IVT: índice de vulnerabilidade à transmissão viral.

Antes da aplicação do modelo ADMC, foi realizada uma análise descritiva das variáveis independentes selecionadas, de modo a evidenciar suas distribuições estatísticas entre os 853 municípios de Minas Gerais. A Tabela 2 apresenta os valores médios, desvios-padrão, medianas, mínimos e máximos observados para cada variável, permitindo visualizar a heterogeneidade estrutural e socioeconômica do estado.

**Tabela 2:** Estatísticas descritivas das variáveis independentes utilizadas na composição do IVTSARS-CoV-2.

Variável	Média	Desvio-padrão	Mediana	Mínimo	Máximo
A-URB	5,2	15,1	1,57	0	287,41
A-COR	5,37	6,07	3,37	0,25	64,42
EFS	7,09	3,97	6,34	0,21	74,78
EFC	2,37	1,68	1,97	0,17	12,08
EFIC	0,39	1,03	0,09	0	11,47
EFIT	2,52	4,09	0,97	0	44,2
IMRS-V	0,54	0,1	0,54	0,29	0,82
IMRS-CEL	0,53	0,19	0,52	0	1
DV	38,22	256,28	10,21	0,16	6.902,97

IVT_SARS-CoV-2_: índice de vulnerabilidade à transmissão do vírus SARS-CoV-2; A-URB: área urbana dos municípios; A-COR: área de corredores viários (ruas e rodovias); EFS: empregos no setor de serviços; EFC: empregos no setor de comércio; EFIC: empregos na indústria da construção; EFIT: empregos na indústria de transformação; IMRS-V: Índice Mineiro de Responsabilidade Social – Vulnerabilidade; IMRS-CEL: Índice Mineiro de Responsabilidade Social – Cultura, Esporte e Lazer; DV: densidade de veículos.

O IVT_SARS-CoV-2_ obtido por meio do modelo de análise multicritério variou de 1 a 8 para os 853 municípios de Minas Gerais (Figura 1). Os municípios que obtiveram um resultado de 1 foram os caracterizados como de menor risco de transmissão da doença. Por outro lado, aqueles que registraram valores mais altos de IVT_SARS-CoV-2_ foram classificados como municípios com maior risco de transmissão da viral.

**Figura 1:** Níveis do IVTSARS-CoV-2 para os municípios de Minas Gerais.

O maior valor do IVT_SARS-CoV-2_ foi registrado em Belo Horizonte (8), seguido de Uberlândia (6). Outros quatro municípios apresentaram índice igual a 5: Uberaba, vizinha de Uberlândia; Contagem e Nova Lima, limítrofes à capital; e Juiz de Fora, no sudeste do estado. A distribuição espacial dos maiores índices revelou concentração de elevada vulnerabilidade em áreas associadas a polos urbanos e regiões metropolitanas, como a Região Metropolitana de Belo Horizonte, Triângulo Mineiro, Sul de Minas e Zona da Mata. Esses territórios apresentam elevada densidade urbana, conectividade viária e setores econômicos com alta interação social – aspectos refletidos nas variáveis A-URB, A-COR, DV, EFS e EFC, de maior peso no modelo. Em contraste, municípios do Norte e Nordeste de Minas Gerais, marcados por menor densidade populacional e infraestrutura urbana e econômica reduzida, apresentaram os menores valores do índice.

Entre os 853 municípios de Minas Gerais, 31 destacaram-se por apresentarem os maiores níveis de vulnerabilidade à transmissibilidade do SARS-CoV-2. Além dos seis municípios previamente mencionados, com índices variando entre 5 e 8, outros 25 municípios atingiram um IVT_SARS-CoV-2_ igual a 4, conforme detalhado na Tabela 3. Esses municípios estão distribuídos em diversas regiões do estado, refletindo a heterogeneidade da vulnerabilidade à covid-19 em Minas Gerais.

**Tabela 3:** Municípios com os maiores valores de IVTSARS-CoV-2, de acordo com o modelo proposto.

Código IBGE	Município	IVT	Código IBGE	Município	IVT
3106200	Belo Horizonte	8	3152501	Pouso Alegre	4
3170206	Uberlândia	6	3104007	Araxá	4
3170107	Uberaba	5	3134202	Ituiutaba	4
3118601	Contagem	5	3127701	Governador Valadares	4
3136702	Juiz de Fora	5	3127107	Frutal	4
3144805	Nova Lima	5	3148103	Patrocínio	4
3143302	Montes Claros	4	3147006	Paracatu	4
3148004	Patos de Minas	4	3131307	Ipatinga	4
3106705	Betim	4	3131703	Itabira	4
3170404	Unaí	4	3120904	Curvelo	4
3122306	Divinópolis	4	3147907	Passos	4
3103504	Araguari	4	3107406	Bom Despacho	4
3170701	Varginha	4	3126109	Formiga	4
3151800	Poços de Caldas	4	3168606	Teófilo Otoni	4
3167202	Sete Lagoas	4	3152105	Ponte Nova	4
3125101	Extrema	4	-	-	-

IVT: índice de vulnerabilidade à transmissão viral; IBGE: Instituto Brasileiro de Geografia e Estatística.

IVT: índice de vulnerabilidade à transmissão viral.

A Figura 2 permite visualizar a vulnerabilidade regional agregada, identificando microrregiões com padrão mais elevado de IVT_SARS-CoV-2_. Microrregiões como Belo Horizonte, Uberlândia, Uberaba e Juiz de Fora revelam forte expressão do dinamismo urbano e da mobilidade regional como fatores de risco. Adicionalmente, é possível observar que parte dos municípios com alto IVT_SARS-CoV-2_ também são sedes de superintendências ou gerências regionais de saúde^40^, o que reforça seu papel como centros articuladores de fluxos territoriais. A espacialização do índice evidencia, portanto, uma lógica de disseminação associada à centralidade urbana e à estrutura de rede regional, aspectos que devem ser considerados em estratégias de prevenção e resposta a pandemias futuras.

**Figura 2:** Níveis do IVTSARS-CoV-2 e microrregiões de maior vulnerabilidade no âmbito de Minas Gerais.

A performance do modelo, avaliada por meio da média dos valores de EQM obtidos em cada iteração da validação cruzada, se mostrou bastante satisfatória. Sendo que o valor médio do EQM foi extremamente pequeno, aproximadamente 5,8331 x 10^32^.

## DISCUSSÃO

A variável de maior peso na ADMC foi A-URB, contribuindo com 22,92% do peso total do modelo. Conforme indicado na Tabela 1, essa variável apresentou a mais alta correlação com o número de casos de covid-19 (r = 0,925). Trata-se de um indicador diretamente associado à densidade de construções e à concentração populacional^41^. O crescimento das áreas urbanas tende a aumentar a exposição das populações a doenças respiratórias, devido à maior proximidade entre indivíduos e à complexidade das redes de contato social^32,42^.

Além disso, centros urbanos atraem fluxos regionais, nacionais e internacionais em função da oferta diversificada de serviços e atividades econômicas^43^. Esse dinamismo urbano pode gerar cenários mais propícios à disseminação viral, especialmente em locais com desigualdade no acesso à saúde e com condições precárias de moradia^9,47^.

A segunda variável de maior importância para a ADMC foi A-COR, sendo o peso dessa variável correspondente a 17,54% do IVT_SARS-CoV-2_. Inicialmente, o aumento de casos de covid-19 se deu por meio da difusão hierárquica e a difusão por contágio adquiriu maior relevância a partir do estabelecimento da transmissão local do vírus SARS-CoV-2^48,49^. Nesse sentido, o deslocamento de indivíduos infectados pelo SARS-CoV-2 passou a exercer um papel crucial no aumento dos casos da doença^50^. Ademais, o deslocamento populacional por meio de transportes públicos revelou-se um fator relevante para a transmissibilidade viral, tanto em virtude da aglomeração de pessoas quanto do tempo de exposição^53,54^.

Em áreas urbanas, a maior conectividade e densidade populacional tendem a facilitar a disseminação rápida de patógenos como o SARS-CoV-2, enquanto as áreas rurais, embora menos densas, podem apresentar vulnerabilidade devido à conectividade com centros urbanos. Adicionalmente, medidas de controle podem apresentar eficácia limitada em áreas urbanas em comparação com áreas rurais^55^. De maneira geral, a densidade populacional e as intensas interações sociais em centros urbanos contribuem significativamente para a rápida disseminação de patógenos, aumentando o risco de surtos e pandemias^56,57^.

Também relacionada à mobilidade, a variável densidade de veículos (DV) apresentou peso de 12,86% no modelo. Para além do deslocamento de pessoas, essa variável tem implicações ambientais importantes. A emissão de poluentes atmosféricos, como o material particulado PM_2,5_ e PM_10_^58,59^, pode comprometer o sistema respiratório e agravar quadros de infecção por covid-19^60,61^. Estudos têm relacionado a poluição atmosférica à elevação de casos e óbitos por covid-19 em diferentes regiões do mundo^62^.

No que diz respeito à estrutura ocupacional dos municípios, destacaram-se as variáveis empregos no setor de comércio (EFC) e empregos no setor de serviços (EFS), com pesos de 11,79% e 8%, respectivamente. Como ilustrado na Tabela 1, esses setores implicam maior interação social e aglomeração, o que aumenta o risco de transmissão viral. Trabalhadores desses setores – especialmente em serviços essenciais, como transporte, alimentação e saúde – foram particularmente vulneráveis durante a pandemia^67^.

Os subíndices IMRS-V e IMRS-CEL corresponderam respectivamente a 9,74 e 8,40% dos pesos para a ADMC. Dessa maneira, temos a condição de vulnerabilidades social sendo positivamente relacionada à maior incidência de covid-19^5,7,70^. Assim como a possibilidade de cidades com mais infraestruturas destinadas a fins recreativos terem promovido a aglomeração de pessoas em detrimento do distanciamento social^68^. Para além disso, no momento de registro dos casos de covid-19 utilizados neste trabalho (fevereiro de 2022), já havia certa tranquilidade e volta à normalidade por parte da população, devido ao avanço das campanhas de vacinação^71^. Além disso, não houve consideração por parte da sociedade de que a variante Ômicron, que estava em circulação no estado, tinha grande potencial de disseminação^27^. Vale ressaltar que a “Emergência em Saúde Pública de Importância Nacional em decorrência da infecção humana pelo novo coronavírus (2019-nCoV)” foi encerrada pela Portaria GM/MS nº 913, de 22 de abril de 2022, e entrou em vigor 30 dias após sua publicação^72^.

De modo geral, as variáveis incluídas no modelo refletem a multiplicidade de fatores associados à vulnerabilidade territorial à covid-19 em Minas Gerais. A distribuição espacial dos valores do IVT_SARS-CoV-2_ evidencia a disseminação do vírus em função da urbanização local, da mobilidade e da estrutura social dos municípios.

Os baixos erros obtidos durante a validação cruzada indicam a consistência estatística e a robustez dos resultados. O índice mostrou-se sensível às variações intraestaduais, revelando padrões territoriais de vulnerabilidade à transmissão do SARS-CoV-2, fortalecendo seu potencial de aplicação em outras doenças transmissíveis e estratégias de prevenção de futuras pandemias.

Entretanto, algumas limitações relacionadas às variáveis independentes se destacam. A maioria dos dados socioeconômicos e estruturais referem-se a 2019, por ser o conjunto mais recente disponível em nível municipal com abrangência estadual. Esse desfasamento temporal, em relação ao período analisado (fevereiro de 2022), pode comprometer parcialmente a atualidade das estimativas, sobretudo diante das mudanças sociais e econômicas aceleradas pela pandemia. Além disso, embora as variáveis tenham sido selecionadas com base em uma literatura consolidada, trata-se de proxies que não captam diretamente aspectos mais dinâmicos, como fluxos populacionais diários ou mudanças comportamentais induzidas por políticas públicas. Outro ponto a considerar é a existência de subnotificações no Brasil, as quais podem afetar a qualidade e a completude dos registros utilizados^73^. Por fim, cabe destacar que os dados são provenientes de diferentes fontes – como Fundação João Pinheiro, MapBiomas e OpenStreetMap – e utilizam metodologias distintas em termos de abrangência e periodicidade, o que pode introduzir heterogeneidade nas variáveis explicativas.

## CONSIDERAÇÕES FINAIS

Este estudo avaliou a vulnerabilidade dos municípios de Minas Gerais à transmissão do SARS-CoV-2 utilizando o índice IVT_SARS-CoV-2_, baseado no modelo de ADMC. O índice evidenciou o papel crucial de variáveis socioeconômicas e de infraestrutura urbana, como mobilidade, densidade de veículos, conectividade e ocupação profissional, na disseminação do vírus. Embora essas variáveis expliquem parte da dinâmica de transmissão, limitações como subnotificação de casos e ausência de fatores comportamentais afetam a precisão do modelo.

Apesar das limitações, como subnotificação de casos e ausência de variáveis comportamentais, o IVT_SARS-CoV-2_ demonstra ser uma ferramenta apropriada para subsidiar políticas públicas, priorizar recursos e mitigar crises sanitárias. Além disso, o índice pode ser aplicado a outras doenças transmissíveis e adaptado a diferentes contextos regionais, provendo análises específicas e alinhadas às necessidades locais.

Os resultados destacam a importância de compreender as dinâmicas socioespaciais que influenciam a vulnerabilidade dos territórios, contribuindo para estratégias mais eficientes de enfrentamento de pandemias. Nossos resultados, de maneira inovadora, mostram a necessidade de incluir corredores e conectividade urbana no contexto do planejamento territorial como estratégia de mitigação a eventos pandêmicos. Estudos futuros que explorem a inclusão de variáveis comportamentais e novas abordagens metodológicas podem aprimorar ainda mais as análises realizadas, aumentando a aplicabilidade e a precisão.

O índice proposto pode ser incorporado como insumo técnico em planos estaduais e municipais de contingência sanitária, bem como em iniciativas de ordenamento territorial voltadas à mitigação de riscos epidemiológicos.

## Data Availability

Os dados estão disponíveis mediante solicitação ao autor correspondente.
